# Dapansutrile in multidisciplinary therapeutic applications: mechanisms and clinical perspectives

**DOI:** 10.3389/fphar.2025.1731165

**Published:** 2025-12-03

**Authors:** Fuwei Bai, Dongyang Wang, Yingying Wu

**Affiliations:** State Key Laboratory of Oral Diseases, National Center for Stomatology, National Clinical Research Center for Oral Diseases, West China Hospital of Stomatology, Sichuan University, Chengdu, China

**Keywords:** dapansutrile, anti-inflammatory, NLRP3 inflammasome, combination drugs, OLT1177

## Abstract

Dapansutrile, an orally active and selective NLRP3 inflammasome inhibitor, exerts its effects by directly binding to the NLRP3 NACHT domain. This action disrupts inflammasome assembly and caspase-1 activation, thereby inhibiting the maturation and release of the pro-inflammatory cytokines IL-1β and IL-18. Beyond this core inhibition, dapansutrile modulates immune cell chemotaxis and inhibits pyroptosis. Preclinical and clinical studies demonstrate its efficacy in mitigating pathology in diverse conditions, including gouty arthritis, cardiovascular diseases, neurodegenerative disorders, inflammatory bowel disease, and periodontitis. A favorable safety profile distinguishes it from other NLRP3 inhibitors like MCC950, with no significant hepatotoxicity reported in trials. Furthermore, dapansutrile exhibits synergistic effects when combined with agents such as lonafarnib or immune checkpoint inhibitors, enhancing anti-inflammatory and anti-tumor responses. This review consolidates evidence on dapansutrile’s molecular mechanisms, therapeutic applications, and biosafety, highlighting its potential as a novel, well-tolerated, and versatile anti-inflammatory agent. Future research should focus on optimizing its delivery, particularly to the central nervous system, and leveraging artificial intelligence to predict effective drug combinations.

## 1 Introduction

Inflammatory diseases pose a significant health burden worldwide, particularly among the elderly population, with conditions such as osteoarthritis being highly prevalent (Kloppenburg et al., 2025). Although currently available anti-inflammatory drugs can alleviate symptoms, their long-term use is often associated with considerable adverse effects (Xie et al., 2023). For instance, non-steroidal anti-inflammatory drugs (NSAIDs), which are widely employed in clinical practice, have been demonstrated in numerous trials to exert detrimental effects on the cardiovascular system, kidneys, and brain (Bindu et al., 2020). Moreover, in chronic inflammatory conditions such as inflammatory bowel disease, patients frequently experience primary or secondary loss of response to biologic agents or small-molecule drugs (Cai Z. et al., 2021). These therapeutic challenges underscore the urgent need for novel anti-inflammatory strategies.

Pro-inflammatory cytokines, including IL-1β and IL-18, play a pivotal role in driving inflammatory responses (Kaminska et al., 2024). The maturation and release of these key cytokines are orchestrated by the NLRP3 inflammasome, a multiprotein complex whose dysregulation contributes to the pathogenesis of a wide range of inflammatory diseases, underscoring its therapeutic relevance (Cabral et al., 2025). The assembly and activation of the NLRP3 inflammasome require a two-signal process: Priming and Activation. The priming signal is initiated when pathogen- or damage-associated molecular patterns bind to pattern-recognition receptors such as Toll-like receptors (TLRs) or TNF receptors (TNFR), subsequently triggering the NF-κB signaling pathway. This priming signal promotes the expression of NLRP3, pro-IL-1β, pro-IL-18, caspase-1, and GSDMD (Paik et al., 2025). The subsequent activation phase then proceeds in two key steps: firstly, NLRP3 associates with ASC and NEK7 to form the inflammasome complex in response to activation stimuli like crystalline substances, ROS, or extracellular DNA. Secondly, this assembly triggers caspase-1 activation, which processes the pro-forms of IL-1β and IL-18 into their active isoforms, facilitating the inflammatory response through their release (Sharif et al., 2019).

Among the compounds targeting NLRP3, MCC950 is one of the most extensively studied inhibitors. It effectively suppresses NLRP3 activation and subsequent inflammatory processes (Coll et al., 2015) (The advantages and limitations of NLRP3 inhibitors are shown in Table 1). However, despite its potent inhibitory effects across multiple disease models, potential hepatotoxicity may limit its clinical translation (Li H. et al., 2022).

**TABLE 1:** The advantages and limitations of NLRP3 inhibitors.

Name	Advantages	Limitations	References
MCC950	One of the most representative NLRP3 inhibitors,has been demonstrated in several animal models	phase I clinical trials confirmed the potential hepatotoxicity	Perera et al. (2018), Muela-Zarzuela et al. (2024)
compound B6	Its IC50 value is 10.69 nM, indicating low toxicity	B6 did not inhibit LPS-induced priming of the NLRP3 inflammasome	Lv et al. (2025)
ZYIL1	Safety profile, rapid absorption, marginal accumulation, and significant inhibition of IL-1β and IL-18 level	The population examined was healthy adult males, the majority of whom self‐selected their race as Asian	Parmar et al. (2023)
GDC-2394	an *in vitro* and *in vivo* safety profile suitable for advancement into human clinical trials	GDC-2394s mechanism of action has not been fully elucidated, and its precursor compound exhibits nephrotoxicity	McBride et al. (2022)
Selnoflast	The drug significantly suppressed plasma IL-1β levels	There were no meaningful differences in the expression of an IL‐1‐related gene signature in sigmoid colon tissue	Klughammer et al. (2023)
DFV890	DFV890 ((R)-1) showed low clearance and steady-state volume of distribution, high plasma protein binding, good oral bioavailability, and good half-life across all four preclinical species tested	Clinical trial data are limited	Shen et al. (2025)
NT-0249	NT-0249 dose-dependently suppressed IL-1β production in response to inflammatory stimuli	When administered at low concentrations with intermittent dosing, the inflammatory markers showed no reduction, suggesting that its efficacy may be influenced by drug dosage and frequency of administration	Doedens et al. (2024)
NT-0796	A selective NLRP3 inflammasome inhibitor with demonstrated *in vivo* brain penetration	No clinical trial data in human subjects are available	Harrison et al. (2023)
Alantolactone	Alantolactone is a naturally derived compound extracted from plants	It might have multiple targets	Li et al. (2023a)

**FIGURE 1:**
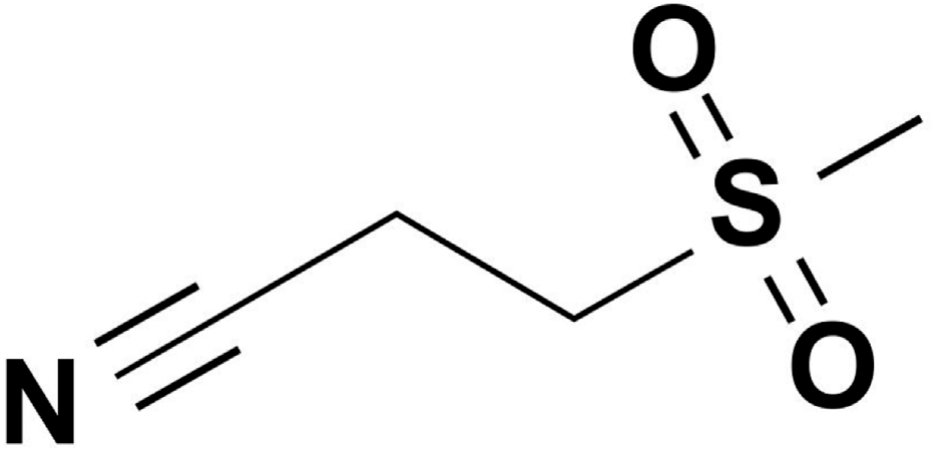
Structure of dapansutrile (OLT1177).

As a selective NLRP3 inflammasome antagonist, dapansutrile is an orally active β-sulfonyl nitrile compound (Figure 1). Preclinical studies have identified it as a targeted NLRP3 inhibitor (Klück et al., 2020). In gout models, dapansutrile has demonstrated favorable safety and efficacy profiles, although mild adverse effects such as metabolic disturbances and gastrointestinal reactions have been reported (Klück et al., 2020). Furthermore, studies indicate that dapansutrile can mitigate the severity of endotoxin-induced systemic inflammation and joint inflammation (Cao et al., 2022). Dapansutrile exerts a pleiotropic anti-inflammatory action that extends beyond direct NLRP3 inflammasome inhibition. It also orchestrates the inflammatory milieu by modulating key cytokines such as IL-1β and IL-6, thereby disrupting the chemotaxis and activation of inflammatory cells (Kiran et al., 2022). These findings support dapansutrile as a promising anti-inflammatory agent and a potential alternative to MCC950. Therefore, a comprehensive summary of its current applications is warranted.

This review systematically elaborates the molecular mechanisms by which dapansutrile acts as a selective NLRP3 inflammasome inhibitor, including its binding to the NLRP3 protein, disruption of inflammasome assembly and activation, and subsequent inhibition of caspase-1 activation and maturation of IL-1β/IL-18. Additionally, its roles in regulating pyroptosis and NF-κB signaling will be examined. Therapeutically, this article summarizes advances in the preclinical and clinical evaluation of dapansutrile for gouty arthritis, rheumatoid arthritis, inflammatory bowel disease, atherosclerosis, and neuroinflammatory conditions, and assesses its potential and challenges as a next-generation anti-inflammatory drug.

## 2 Mechanism of action of dapansutrile

### 2.1 Targeted inhibition of the NLRP3 inflammasome

The selective inhibition of the NLRP3 inflammasome by dapansutrile stems from its direct interaction with the NLRP3 protein, disrupting its assembly process. Structural insights from bioinformatics analyses reveal that dapansutrile anchors into the NACHT domain, which suppresses the domain’s ATPase function and thereby impedes the oligomerization of NLRP3 (Cao et al., 2022). This action effectively suppresses the formation of inflammasome specks, which are aggregates of components such as NEK7, NLRP3, and pro-IL-1β. (Kiran et al., 2022). In addition to directly binding NLRP3, Dapansutrile impedes inflammasome assembly through other binding mechanisms. By fluorescent resonance energy transfer (FRET) analysis, the researchers found that Dapansutrile reduced NLRP3 binding to ASC and Caspase-1. Importantly, dapansutrile exhibits high specificity for NLRP3 and does not influence the activation pathways of other inflammasomes, including AIM2 and NLRC4 (Marchetti et al., 2018a). This specificity may be due to the fact that Dapansutrile has no effect on the upstream signals that initiate inflammasomes, such as K+ efflux and P2X7 receptor. However, the effects of Dapansutrile on other NLRP family members or the pyrin inflammasome remain unclear. Here, we have also outlined the advantages and limitations of other NLRP3 inflammasome inhibitors currently under investigation.

**FIGURE 2:**
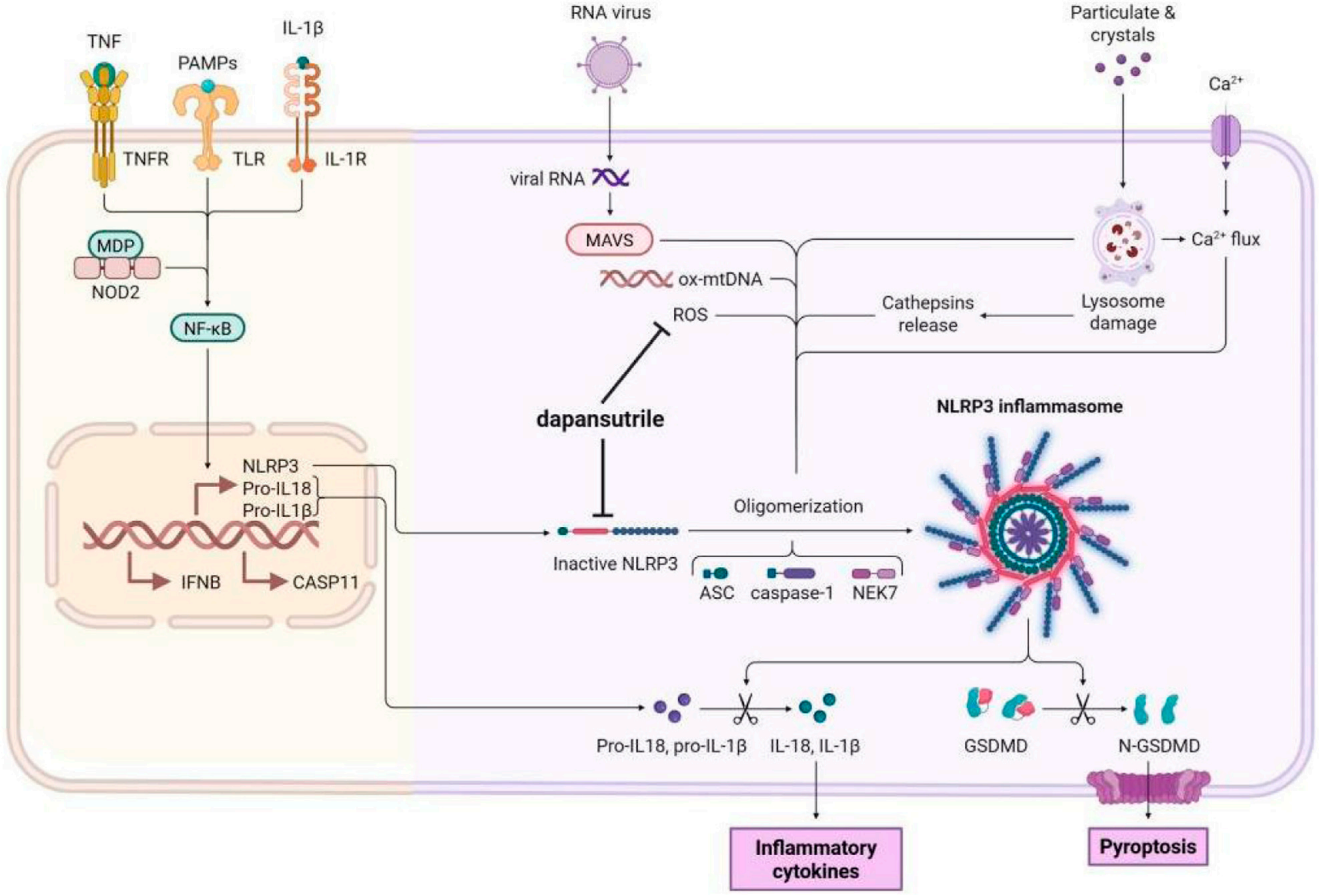
Mechanism of Dapansutrile on NLRP3 inflammasome inhibition. The release of NLRP3 inflammasome includs a two-step process: priming and activation. During the activation phaese, Dapansutrile directly binds to the inactive NLRP3 protein and disruption of inflammasome assembly. It also reduces the ROS production to inhibit the oligomerization. The figure was created in biorender.

Figure 2: Mechanism of Dapansutrile in NLRP3 Inflammasome Inhibition. NLRP3 inflammasome activation proceeds through two sequential stages: priming and activation. During the activation phase, Dapansutrile exerts its inhibitory effect by: (1) directly binding to the inactive NLRP3 protein, thereby preventing inflammasome assembly; and (2) reducing ROS production to suppress NLRP3 oligomerization. The figure was created in biorender.

**FIGURE 3:**
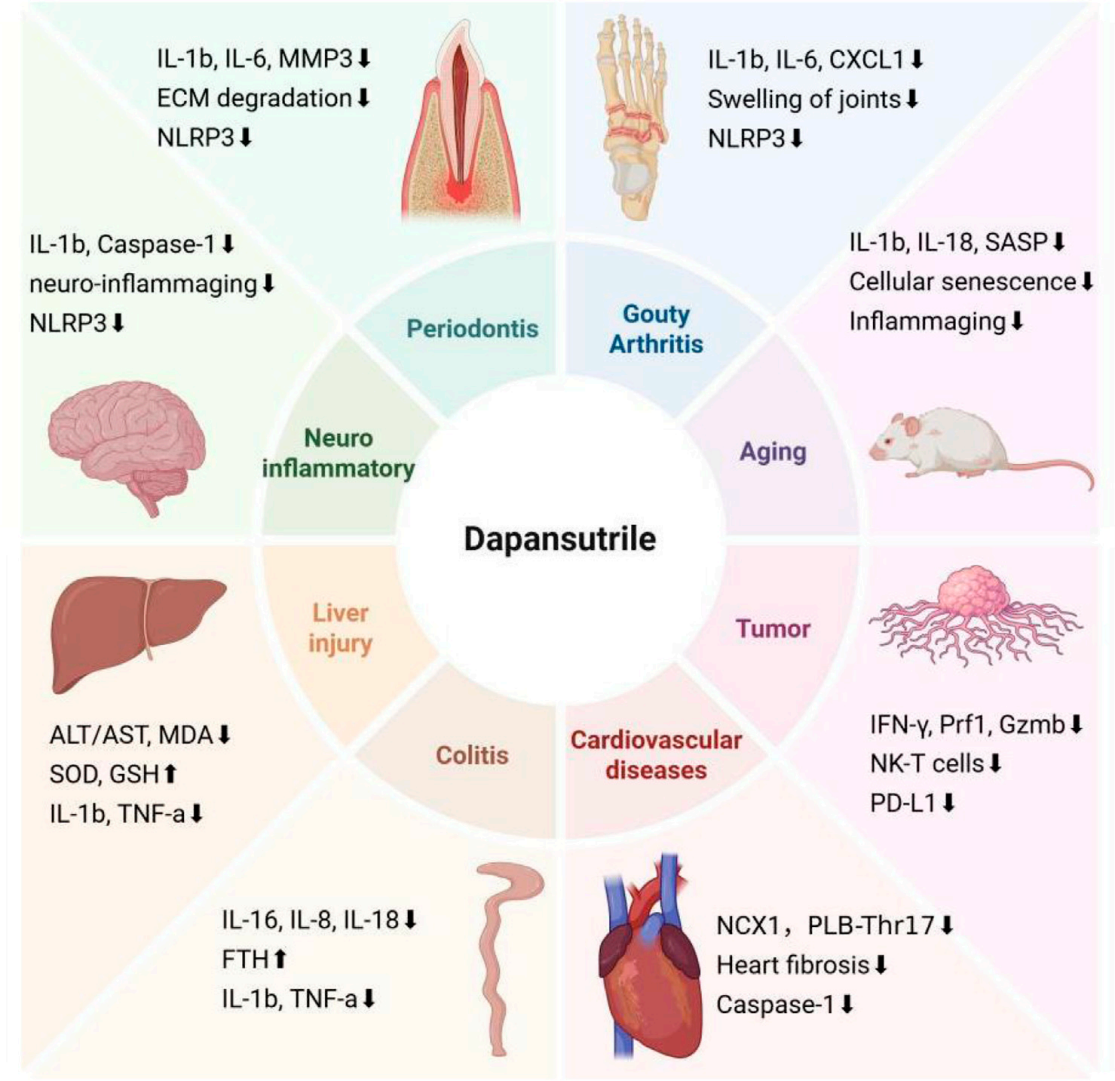
Targets of Dapansutrile on distnct diseases. In addition to regulating NLRP3 inflammation, dapansutrile also targets different systemic diseases by regulating a variety of inflammatory factors and cytokines: Gouty arthritis, Aging, Tumor, Cardiovascular diseases, Colitis, Liver injury, Neuroinflammatory diseases, and Periodontis. The figure was created in biorender.

### 2.2 Regulation of immune cells chemotaxis

Beyond direct NLRP3 inhibition, dapansutrile has been shown to modulate neutrophil chemotaxis. Studies indicate that it can interfere with the recruitment and activation of inflammatory cells. In a gouty arthritis model, dapansutrile alleviated local joint inflammation by reducing neutrophil infiltration to the site of inflammation (Marchetti et al., 2018b). This dual mechanism, simultaneously targeting inflammasome activation and immune cell migration, confers dapansutrile a unique therapeutic advantage in inflammatory diseases. Part of this activity may stem from its direct interaction with proinflammatory factors. A reported mechanism involves dapansutrile forming a stable complex with IL-1β, exerting a dual regulatory role in osteoblasts. It not only suppresses IL-1β–dependent pathways (evidenced by reduced COX2, iNOS, and MMP3) but also promotes the expression of anti-inflammatory and pro-regenerative mediators (IL-10, SOX9, COL2), thereby tilting the cellular balance toward repair (Tang et al., 2022). These findings suggest that dapansutrile modulates inflammation both directly at the cytokine level and indirectly through immune cell regulation. Additionally, besides neutrophils, dapansutrile can inhibit tissue infiltration by T cells and mast cells, thereby ameliorating disease severity in models such as interstitial cystitis (Kiran et al., 2022).

### 2.3 Mechanism of pyroptosis intervention

The anti-pyroptotic effect of dapansutrile represents another key anti-inflammatory mechanism. By suppressing NLRP3 inflammasome activation, it inhibits caspase-1–mediated cleavage of gasdermin D (GSDMD), ultimately preventing pyroptosis (Cai Y. et al., 2021). Pyroptosis is a programmed cell death mediated by GSDMD, which is characterized by the formation of plasma membrane pores and the release of inflammatory factors. Pyroptosis is necessary for the killing of bacteria and cancer cells. However, excessive pyroptosis leads to pathological activation of the inflammatory response (Bai et al., 2025). In an Alzheimer’s disease model, NLRP3-driven pyroptosis contributed significantly to disease progression, and dapansutrile ameliorated pathological changes via this pathway. Similarly, in a liver injury model, dapansutrile attenuated tissue damage by reducing ROS production and interrupting the ROS–NLRP3–pyroptosis axis (Fan et al., 2023). This anti-pyroptotic activity has been confirmed across multiple cell types, including microglia, airway epithelial cells, and hepatocytes (Zhang et al., 2024).

### 2.4 Mechanism of combination drugs

Dapansutrile also demonstrates notable synergistic effects when combined with other agents. For example, in a progeria model, co-administration with the farnesyltransferase inhibitor lonafarnib significantly improved inflammatory and aging phenotypes, extended survival, and alleviated progeria-like defects more effectively than lonafarnib alone (Muela-Zarzuela et al., 2024). Mechanistic studies suggest that this combination acts through concurrent inhibition of the NLRP3 inflammasome and protein farnesylation signaling. Notably, the two drugs jointly affect key inflammatory mediators such as HRAS, STAT3, JUN, and IL-6, forming a distinctive interaction network.

The complementary mechanisms of dapansutrile and other anti-inflammatory drugs support multi-level therapeutic synergism. For instance, its action complements that of p38 MAPK pathway inhibitors such as methotrexate (Alenezi et al., 2025). In the present study, dapansutrile similarly reduced the expression of inflammatory regulatory signals (CD4, CD8, IFN-γ, and JNK), although failed to completely restore them to normal levels. The regulation of inflammatory signaling by dapansutrile suggests potential combined applications with other anti-inflammatory molecules. Systems pharmacology analyses indicate that such combinations can produce enhanced anti-inflammatory outcomes by concurrently modulating processes like AKT phosphorylation and inflammasome activation. Metabolic pathway analyses further suggest that dapansutrile can complement the effects of glucocorticoids or NSAIDs through coordinated regulation of drug metabolism pathways (Dinarello et al., 2023). Specifically, dapansutrile inhibited the phosphorylation level of STAT3 Y705 as well as serine 727, thereby affecting the regulation of STAT3 on target genes as well as mitochondria. The combination of dapansutrile and dexamethasone reduced the ATP and glycolysis levels of the tumor cells, thus exerting a synergistic therapeutic effect. Overall, these combination strategies not only enhance therapeutic efficacy but may also allow dose reduction of individual drugs, thereby mitigating adverse effects.

## 3 Therapeutic applications in inflammatory diseases

### 3.1 Protective effects in gouty arthritis

To evaluate the concentration-dependent therapeutic efficacy of OLT1177, varying doses (60, 200, and 600 mg/kg) achieving plasma levels of 9.22 ± 0.55, 16.73 ± 9.89, and 41.4 ± 3.68 μg/mL, respectively, were administered in an MSU-induced gouty arthritis of murine model. The results established a direct correlation between the systemic exposure of OLT1177 and the magnitude of reduction in joint inflammation. Pretreatment with an OLT1177-enriched diet for 3 weeks prior to MSU challenge effectively suppressed the synovial expression of Nlrp3 and its downstream target Il1b. This molecular-level inhibition translated into a significant reduction in synovial IL-1β protein and, consequently, markedly less severe joint swelling compared to the standard diet group. These findings indicate that dapansutrile selectively inhibits the NLRP3 inflammasome, markedly alleviating joint swelling and improving motor dysfunction (Klück et al., 2020; Marchetti et al., 2018b). A phase 2a study enrolled 29 patients in the per-protocol population, with doses of 100, 300, 1000, and 2000 mg/day. Notably, plasma levels of IL-1β showed a decreasing trend across all groups, though statistical analysis revealed no intergroup differences. Significant reductions in plasma IL-6 concentrations were observed on day 7 in the 2000 mg/day, 1000 mg/day, and 300 mg/day groups (p < 0.05). In gout, elevated IL-6 levels may serve as a marker for active IL-1β, as the ability of IL-1β to induce IL-6 is well-established. Elevated IL-6 is commonly used as a surrogate marker for sub-picogram levels of IL-1β in humans. Concurrent with decreased IL-6 levels, most cohorts exhibited significant pain reduction in the target joint as early as day 3, with the 300 mg group demonstrating a mean pain reduction of 68.4% (p = 0.016). By day 7, all dose groups showed significant efficacy, with mean pain reduction rates ranging from 82.1% to 88.9% (Marchetti et al., 2018b).

Assessment of circulating cytokines during dapansutrile treatment revealed a distinct profile: a significant decline in plasma IL-6, no change in TNFα (an NLRP3-independent cytokine), and a non-significant reduction in IL-1β (Klück et al., 2020). The physiological relevance of monitoring IL-6 was further corroborated by a larger clinical study of 500 healthy volunteers, which demonstrated a strong correlation between plasma IL-6 and IL-1β levels, validating IL-6 as a proxy for IL-1β (Ter Horst et al., 2016). These results demonstrate that dapansutrile effectively alleviates target joint pain and local/systemic inflammation during acute gout flares, highlighting its potential as a novel NLRP3 inflammasome-targeted inhibitor for treating gout attacks and other NLRP3-mediated diseases.

### 3.2 Suppression of bone destruction in periodontitis

Studies have shown that the NLRP3 inhibitor MCC950 significantly alleviates ligature-induced periodontitis, reduces IL-1β activation and osteoclast differentiation, and thereby inhibits alveolar bone resorption (He et al., 2020). Similarly, dapansutrile, via selective NLRP3 inflammasome inhibition, demonstrates potential in mitigating periodontitis-related bone loss (Marchetti et al., 2018a). Its mechanism involves multi-target regulation, including suppression of pro-inflammatory cytokines such as IL-1β and IL-6, as well as downregulation of matrix metalloproteinases including MMP3 (Tang et al., 2022). Preclinical studies indicate that dapansutrile not only attenuates inflammatory responses but also preserves bone structure by modulating extracellular matrix degradation (Yip et al., 2024). Notably, dapansutrile exhibits a synergistic effect when combined with lonafarnib, providing additional benefits in improving bone metabolic abnormalities in progeria animal models (Fu et al., 2025). This offers a theoretical basis for developing novel combination therapies targeting bone resorption in periodontitis.

### 3.3 Protective effects in neuroinflammatory diseases

In the field of neurodegenerative diseases, dapansutrile demonstrates potential in modulating neuro-inflammaging (Murda et al., 2022). Studies indicate that age-related chronic low-grade inflammation is a significant risk factor for neurodegenerative disorders such as Alzheimer’s disease (AD) (Abbatecola et al., 2024). The therapeutic potential of NLRP3 inflammasome inhibition by dapansutrile was demonstrated in a model of Alzheimer’s disease in mice. When six-month-old APP/PS1 mice were maintained for 3 months on an OLT1177-enriched diet, their age-dependent cognitive deficits were completely reversed. This was evidenced by a full rescue of learning and memory impairments in the Morris water maze test (P = 0.008 vs. untreated APP/PS1), which was accompanied by a significant restoration of synaptic plasticity (P = 0.007 vs. untreated APP/PS1). Furthermore, NLRP3 inhibition via OLT1177 reduced microglial activation (P = 0.07) and decreased cortical plaque burden (P = 0.03), highlighting its therapeutic potential for neuroinflammation (Lonnemann et al., 2020). However, the therapeutic effects of OLT1177 in humans remain unclear, and existing animal studies cannot definitively demonstrate that Dapansutrile has an absolute curative effect on Alzheimer’s disease. The recovery outcomes in advanced-stage subjects require further investigation. Thus, the efficacy of OLT1177 in late-stage Alzheimer’s disease may become a key objective for future research, which would provide a theoretical foundation for the clinical translation of this drug. Notably, OLT1177 reduced nuclear deformation in Hutchinson-Gilford progeria syndrome (HGPS) fibroblasts, attenuated senescence markers, and improved survival in *Lmna*G609G/G609G mice. Western blot and sequencing analyses showed that *in vivo* administration lowered NLRP3, active caspase-1, and IL-1β protein levels in cardiac and hepatic tissues of *Lmna*G609G/G609G models, suggesting neuroprotection via modulation of the central nervous system’s inflammatory microenvironment (Muela-Zarzuela et al., 2024). Neuroinflammation also plays a pivotal role in Parkinson’s disease (PD) pathology (Qian et al., 2010). Ellagic acid (EA), a natural polyphenol with neuroprotective effects in neurological disorders (Moura et al., 2015), suppresses NLRP3 inflammasome signaling and proinflammatory cytokine release in microglia. However, tyrosine hydroxylase (TH)-positive neuron counts and TH protein assays revealed no direct neuroprotection for dopaminergic neurons (He et al., 2020). The therapeutic potential of OLT1177 for neurodegenerative disorders is supported by evidence from MPTP-induced PD models, which shows that the inhibitor effectively crosses the blood-brain barrier, enabling it to reach concentrations that block NLRP3 assembly in the brain. Subsequent *in vitro* and *in vivo* animal experiments showed OLT1177 significantly reduced α-synuclein monomer/oligomer levels by enhancing microglial autophagy, thereby degrading α-synuclein, lowering proinflammatory cytokines, and protecting dopaminergic neurons and astrocytes (Amo-Aparicio et al., 2023).

In drug-induced experimental autoimmune encephalomyelitis model in mice, OLT1177-enriched chow reduced spinal cord levels of TNFα, CXCL-1, and IL-6 without affecting anti-inflammatory IL-10. Remarkably, OLT1177 also diminished spinal proinflammatory cytokines, immune cell infiltration, and prevented demyelination (Sanchez-Fernandez et al., 2019). While limited data exist on its BBB penetration, its systemic anti-inflammatory efficacy underscores the importance of developing targeted delivery systems for neuroinflammatory diseases.

### 3.4 Potential in attenuating age-related inflammation (inflammaging)

Dapansutrile emerges as a promising candidate for modulating inflammaging, a core pathological process in age-related diseases characterized by low-grade chronic inflammation (Koppula et al., 2021). The condition manifests as a breakdown in inflammatory homeostasis, with sustained elevations in key mediators such as IL-1β and IL-18 (Giunta et al., 2022; Muller and Di Benedetto, 2024). When combined with the existing anti-aging drug lonafarnib, dapansutrile exhibits synergistic anti-inflammatory effects, more effectively reducing tissue inflammation levels and cellular senescence markers while blocking the production of senescence-associated secretory phenotype (SASP). This combination has been shown to significantly extend lifespan and ameliorate multiple aging-related defects in progeroid animal models (Muela-Zarzuela et al., 2024). This combined strategy provides novel insights for intervening in age-related conditions such as cardiovascular aging (Zeng et al., 2024) and metabolic disorders (Yuan et al., 2018), with its safety and efficacy preliminarily validated in Phase II clinical trials (Lonnemann et al., 2020).

## 4 Therapeutic strategies in tumor immunoregulation

Dapansutrile demonstrates unique potential in the field of tumor therapy. Studies have shown that upon activation by various stimuli, EBV can drive the NLRP3 inflammasome into a proliferative phase, while Dapansutrile suppresses NLRP3 transcription, thereby inhibiting inflammasome activation and EBV lytic phase induction (Borde et al., 2023). Dapansutrile enhances anti-tumor immunity by restricting the expansion of myeloid-derived suppressor cells (MDSCs), an effect mediated through its selective inhibition of the NLRP3 inflammasome in tumor cells. This action contributes to reprogramming the immunosuppressive tumor microenvironment (Borde et al., 2023). Notably, the combination of Dapansutrile with anti-PD-1 antibodies markedly improves anti-tumor efficacy. By inhibiting the NLRP3 inflammasome in mice, this combination therapy further activates T-cell immune responses. Specifically, when CD8^+^ T cells were isolated from tumors and assessed for interferon-gamma (Ifng), perforin (Prf1), and granzyme B (Gzmb) gene expression, the dual-inhibitor treatment doubled Ifng expression (P < 0.01), with similar trends observed for Prf1 and Gzmb (Tengesdal et al., 2021).

As a specific NLRP3 inhibitor, Dapansutrile effectively suppresses the inflammasome pathway via oral administration, thereby remodeling the tumor microenvironment (TME). Research in diffuse large B-cell lymphoma identified NLRP3 inflammasome activation as a key driver of immune resistance, demonstrating its role in elevating PD-L1 levels and diminishing cytotoxic T-cell proportions - revealing a promising therapeutic target.An animal studies have demonstrated that dapansutrile, by blocking NLRP3, downregulated PD-L1 expression *in vivo*, diminished PD-1/TIM-3-expressing exhausted T cells, and consequently improved anti-tumor immunity while suppressing tumor growth (Lu et al., 2021). Similarly, after NLRP3 inflammasome activity was inhibited, IL-1β secretion decreased, attenuating inflammatory cascades in the TME, which helps reduce pro-tumorigenic inflammation in the tumor microenvironment (Chen et al., 2018).

Dapansutrile exhibits distinct advantages in tumor immunomodulation, offering novel insights for the development of future anti-tumor drugs.

## 5 Cardiovascular diseases

In patients with stable heart failure and reduced ejection fraction (HFrEF), dapansutrile produced rapid improvements in cardiac function and exercise capacity within 14 days. Treatment elevated LVEF by 5 percentage points (31.5%–36.5%) and prolonged exercise duration by 46 s, all with a favorable biosafety record (Wohlford et al., 2020). At the molecular level, in a rat model of atrial fibrillation (AF) induced by HFrEF, Dapansutrile was found to reduce atrial fibrosis and inflammatory responses while enhancing sarcoplasmic reticulum calcium load during cardiomyocyte calcium transients. It upregulated SERCA2 protein expression and decreased sodium-calcium exchanger (NCX1) and phosphorylated phospholamban (PLB-Thr17) levels, thereby improving calcium homeostasis and myocardial contractile function (Yang et al., 2024). Dapansutrile conferred profound cardioprotection in a murine model of ischemia-reperfusion injury. Administration within the critical 60-min post-reperfusion window reduced myocardial infarct size by 71%, an effect associated with suppressed cardiac Caspase-1 activity and the prevention of subsequent left ventricular systolic dysfunction (Toldo et al., 2019). In a murine model of severe ischemic cardiomyopathy induced by non-reperfused anterior wall myocardial infarction, OLT1177 treatment preserved key cardiac functions. This included significantly better contractile reserve (isoproterenol-induced LVEF increase: +33 ± 11% to +40 ± 6%), maintained β-adrenergic responsiveness, and concurrent prevention of left ventricular diastolic dysfunction (Aliaga et al., 2021). However, some studies indicate that the myocardial infarction models used can induce heart failure in mice (Riehle and Bauersachs, 2019; Salloum et al., 2014), and these studies did not evaluate chronic symptoms of heart failure (e.g., pulmonary congestion). Current clinical and preclinical evidence suggests its potential as a novel therapeutic strategy for heart failure and related conditions, but further in-depth research in cardiovascular diseases remains necessary for validation and refinement.

## 6 Protective effects in multiple organ systems

### 6.1 Joint and cartilage protection

Research has demonstrated that intra-articular injection of Dapansutrile in a rat osteoarthritis (OA) model significantly reduced Mankin and OARSI scores (joint degeneration evaluation metrics). A multi-level analysis revealed the broad impact of Dapansutrile on inflammatory and catabolic pathways. At the RNA level, it modulated key markers (Cox-2, iNOS, Mmp-3/9/13, IL-10), findings that were coherently confirmed at the protein level for COX-2, MMP-3/9/13, SOX-9, and COL2. Furthermore, Western blot analysis demonstrated that this regulatory effect involved the significant suppression of the MAPK signaling pathway, as evidenced by reduced phosphorylation of its key components ERK, JNK, and p38 (Cargnello and Roux, 2011). Specifically, Dapansutrile inhibited phosphorylated ERK and p38 expression, thereby attenuating MAPK pathway activation. This suppression led to reduced expression of matrix metalloproteinases (MMP3, 9, 13) and reversed the IL-1β-induced downregulation of type II collagen (COL2) and chondrogenic markers (SOX9, COL2), highlighting its anti-degenerative effects (Tang et al., 2022). Cartilage degeneration is often associated with TLR/NF-κB pathway activation (Yin et al., 2025). Dapansutrile’s inhibition of NLRP3 may indirectly regulate these pathways, suggesting its potential as a novel therapeutic strategy for joint and cartilage disorders.

### 6.2 Hepatoprotective effects

In hepatitis-induced liver injury in mice, treatment with Dapansutrile was found to significantly reduce ALT/AST levels and ameliorate histopathological damage in liver tissue. Mechanistic studies revealed that the drug alleviates oxidative stress by increasing glutathione (GSH) and superoxide dismutase (SOD) levels while reducing malondialdehyde (MDA) levels. The observed reduction in critical inflammatory mediators, including NLRP3, TNF-α, IL-6, and IL-1β, is consistent with Dapansutrile acting through a mechanism that centrally involves the inhibition of key inflammatory pathways (Alenezi et al., 2025). In chemical-induced liver injury in mice, Dapansutrile suppresses the NLRP3 inflammasome, further reducing serum transaminases, IL-1β, and TNF-α expression levels. It also inhibits the cleavage of Caspase-1, IL-1β, and gasdermin D (GSDMD), improving DMF-induced infiltration of hepatic macrophages and neutrophils, thereby significantly attenuating liver injury (Zhang et al., 2024). In multiple clinical trials, Dapansutrile has demonstrated good safety and low hepatotoxicity (Wohlford et al., 2020). Notably, another NLRP3 inhibitor, MCC950, was found to pose potential hepatotoxicity risks in both animal studies and clinical trials, leading to the termination of its Phase I clinical program (Chen et al., 2023). In contrast, available reports have not indicated evidence of hepatotoxicity for Dapansutrile in animal models; instead, it has demonstrated protective effects against chemically-induced (e.g., Con A) and progeria-associated liver injury. Its favorable safety profile may stem from its specific inhibition of the NLRP3 inflammasome without interfering with other hepatic metabolic pathways (Muela-Zarzuela et al., 2024). Furthermore, Dapansutrile may serve as an adjunctive therapy in combination with lonafarnib for treating Hutchinson-Gilford progeria syndrome (HGPS), with mechanisms including reducing inflammation and delaying senescence-related phenotypes (Muela-Zarzuela et al., 2024), which indirectly supports its potential in treating chronic liver diseases.

### 6.3 Intestinal protection

Dapansutrile (OLT1177) demonstrates dual therapeutic effects in dextran sodium sulfate (DSS)-induced murine colitis models. Firstly, it significantly suppresses macrophage infiltration into damaged colonic mucosa and epithelial layers while reducing serum levels of pro-inflammatory cytokines (IL-1β, IL-6, IL-8, IL-18, TNF-α). Secondly, it upregulates ferritin heavy chain (FTH) expression, whose ferroxidase activity converts toxic Fe^2+^ to Fe^3+^ (Ferreira et al., 2001), thereby alleviating colonic iron overload and macrophage-mediated inflammation, a mechanism also linked to NLRP3 inhibition (Yu et al., 2025).

Notably, combination therapy with OLT1177 and BBG (a P2X7R blocker) synergistically reduces leukocyte infiltration, colon wall thickness, and improves histopathological scores. This regimen normalizes expression levels of NF-κB, IL-6, TNF-α, P2X7R, and NLRP3 in mice, while decreasing caspase-1, myeloperoxidase (MPO) activity, and caspase-3 expression. Intriguingly, OLT1177 monotherapy paradoxically elevates caspase-3 and MPO activity (Saber et al., 2021), suggesting complementary mechanisms between NLRP3 and P2X7R inhibition for colitis treatment.

These findings collectively underscore dapansutrile’s promise as a therapeutic target for inflammatory bowel diseases, highlighting its considerable potential for clinical translation.

### 6.4 Renal protection

Chemical renal toxicity is common, with limited therapeutic interventions available. In folic acid (FA)-induced acute kidney injury (AKI) in mice, DAPA injection improved renal tissue integrity, as evidenced by reduced glomerular tuft atrophy and necrosis accompanied by decreased interstitial inflammatory cell infiltration, ameliorated tubular dilation and necrosis, as well as diminished CD86-positive cell infiltration and lower fibrosis percentage. Functional biomarker analysis revealed significant reductions in caspase-1, IL-1β, and IL-18 (p < 0.05). The therapeutic relevance of DAPA was further evidenced by its reduction of Ki-67 (proliferation) and LC-3II (autophagy) (Elsayed et al., 2021). This supports a model where DAPA mitigates FA-induced nephrotoxicity not only by disrupting the core inflammasome/caspase-1/IL signaling axis but also by coordinately regulating associated pathways of renal regeneration and autophagy.

By targeting the NLRP3 inflammasome, dapansutrile inhibits pro-inflammatory cytokine release, modulates oxidative stress, and regulates extracellular matrix degradation. Consequently, it attenuates inflammatory responses in tissues and has demonstrated direct or indirect protective effects across multiple organ models, including the heart, joints, liver, and intestines (Detailed effects are shown in Table 2). Clinical studies in heart failure and osteoarthritis have confirmed its safety and preliminary efficacy (Klück et al., 2020; Wohlford et al., 2020). A comprehensive understanding of the mechanisms behind its organ protection awaits further study.

**TABLE 2:** The targets and effects of Dapansutrile in multidisciplinary therapy.

Diseases	Target	Effect	References
Gouty Arthritis	Synovial tissue	Reduce joint swelling, cellular influx and pain	Klück et al. (2020), Perera et al. (2018)
Periodontis	Osteoclast and osteoblast	Suppression of inflammatory responses and preserves bone structure	Marchetti et al. (2018b), Tang et al. (2022)
Neuro inflammatory	Microglial and fibroblast	Reduce microglial activation and enhanced microglial autophagy	Fan et al. (2023), Zhang et al. (2024), Alenezi et al. (2025), Dinarello et al. (2023), Ter Horst et al. (2016)
Inflammaging	Fibroblast	Extend lifespan and ameliorate multiple aging-related defects	Abbatecola et al. (2024)
Tumor immunoregulation	MDSCs, CD8^+^ T cell	Enhance anti-tumor immune responses	Moura et al. (2015)
Cardiovascular diseases	Cardiovascular fibroblast	Reduce atrial fibrosis and inflammatory responses	Giunta et al. (2022)
Liver protection	Hepatic macrophages and neutrophils	Reduce ALT/AST levels and increase GSH and SOD levels	McBride et al. (2022)
Intestinal protection	Macrophage and leukocyte	Upregulate FTH expression and reduce colon wall thickness	Wohlford et al. (2020), Yang et al. (2024), Toldo et al. (2019)
Renal protection	CD86-positive cel	Reduce glomerular tuft atrophy and modulates oxidative stress	Giunta et al. (2022), Aliaga et al. (2021)

## 7 Biosafety monitoring strategies

For patients with chronic inflammatory conditions such as gouty arthritis and cardiovascular diseases requiring long-term medication, a multi-level monitoring approach is recommended.

1) Basic Monitoring: Routine liver and kidney function tests and complete blood counts, with particular attention to possible changes in white blood cell counts (Song et al., 2024). 2) Targeted Monitoring: Dynamic assessment of NLRP3 pathway-specific markers such as IL-1β and IL-18 (Sanchez-Fernandez et al., 2019). 3) Organ-Specific Monitoring: Periodic evaluation of tissue-specific indicators, including joint cartilage metabolism markers (MMP3, MMP13) and cardiovascular function (left ventricular systolic performance) (Cai Y. et al., 2021; Giunta et al., 2022). For cartilage protection effects observed in animal models (reductions in Mankin and OARSI scores), long-term imaging follow-up of joint structures in patients is advised.

In diseases requiring combination therapy, safety monitoring should also consider drug–drug interactions. When dapansutrile is co-administered with lonafarnib—despite synergistic benefits in improving progeria phenotypes (e.g., reduced kyphosis and inflammatory markers) (Li B. et al., 2022)—specific drug-metabolizing enzymes should be included in the monitoring scheme. For combination regimens such as azathioprine with allopurinol in inflammatory bowel disease (Belhocine et al., 2021), therapeutic drug monitoring (TDM) is recommended, with particular focus on hepatic metabolic enzyme activity to minimize potential liver injury.

## 8 Future research directions

### 8.1 Development of drug delivery systems (e.g., nanocarriers)

Current research indicates that polymer-based drug delivery systems (PDDS) play a crucial role in controlled drug release, though their complex structures and multiple influencing factors limit therapeutic outcomes with conventional methods (Han et al., 2016; Aghajanpour et al., 2025). Nanomicellar systems have been validated as effective carriers for combined anti-inflammatory and antioxidant therapies (Li et al., 2022c), offering valuable insights for optimizing dapansutrile delivery. AI-assisted nanocarriers also show unique advantages; integrated machine learning methods such as random forest and XGBoost genetic algorithms can effectively predict drug release behavior and guide the development of novel delivery systems (Khodadadi et al., 2025). Particularly, chemometrics-based predictive models have been successfully applied to optimize drug delivery platforms, significantly accelerating translation from bench to bedside (Perni and Prokopovich, 2020). Future studies should focus on developing targeted, activatable, and fluorescence imaging-guided nano-delivery systems for dapansutrile (Song et al., 2024), while leveraging molecular modeling and machine learning to optimize formulation design (Li et al., 2022d).

### 8.2 AI-predicted drug combinations

Artificial intelligence holds great potential in predicting combination therapies, especially for complex multi-drug regimens (Zhang et al., 2025). Integrated AI and physiologically based pharmacokinetic (PBPK) platforms can rapidly predict *in vivo* drug efficacy and related uncertainties based solely on molecular structures, and have successfully forecasted eight key drug properties (solubility, pKa, crystal density, intrinsic dissolution rate, apparent permeability, unbound fraction, plasma clearance, and tissue partition coefficients for 15 organs) (Wang et al., 2025). Computational approaches such as Gaussian process regression, combined with active learning and fine grid optimization, can predict the therapeutic effects of dual-drug-loaded nanoparticles with high efficiency using only 25% of experimental data (Jin et al., 2025). Existing studies show that machine learning models excel in predicting drug synergy and antagonism; predictive models integrating multi-source biological data provide a theoretical foundation for precision medicine (Lin et al., 2025). For optimizing dapansutrile-based combinations, perturbation theory–machine learning (PTML) models—already applied in screening drug–vitamin nano-systems for cancer combination therapy (Santana et al., 2019)—can be employed. Future research should emphasize the development of integrated algorithmic models capable of simultaneously predicting drug release profiles and synergistic efficacy (Bannigan et al., 2023), and establish specialized databases for predicting multi-drug combinations in inflammatory diseases (Chen et al., 2023).

## 9 Discussion

Dapansutrile (OLT1177) is defined by its novel, specific NLRP3 inflammasome inhibition, demonstrating consistent mechanisms and broad therapeutic applicability across inflammatory diseases, including gouty arthritis (Marchetti et al., 2018b), neurological disorders (Murda et al., 2022), and cardiovascular diseases (Wohlford et al., 2020). Its core mechanism consistently involves inhibiting the activation or assembly of the NLRP3 inflammasome, leading to reduced release of pro-inflammatory factors such as IL-1β. This ultimately attenuates inflammatory responses and fibrosis by decreasing M1 macrophage polarization and inhibiting pyroptosis. Studies have found that hyperuricemia exacerbates periodontitis and gouty arthritis by activating NLRP3 (Wu et al., 2025; Sun et al., 2025), suggesting a close link between metabolic abnormalities and inflammation, and indicating that NLRP3 inhibitors could be therapeutic for such conditions. In progeria and tumor immunotherapy, OLT1177 ameliorates cellular senescence and promotes tissue repair by reducing progerin secretion (Muela-Zarzuela et al., 2024) and improving the tumor microenvironment (Lu et al., 2021), respectively. Serving as a critical mechanism for controlling inflammasome activity, NLRP3 ubiquitination inhibits both oligomerization and subcellular translocation, thereby preventing its activation (Xu et al., 2022) and highlighting the value of targeted inhibitor development.

The efficacy of OLT1177 across pathologically distinct conditions positions the NLRP3 inflammasome as a central pathogenic driver, serving as a common regulatory node in diverse disease networks. Its selective inhibition thus represents a strategy for normalizing dysregulated inflammatory responses transcending conventional disease boundaries. MCC950 is the most commonly used NLRP3 inhibitor in non-clinical models and has shown efficacy in over 100 inflammatory disease models, but it has not yet received FDA approval (Cabral et al., 2025). This may be due to concerns that its furan group could be associated with drug-induced liver injury (Li Z. et al., 2023) and its incomplete inhibitory effects and complex mechanisms potentially limit its clinical application. Therefore, developing NLRP3 inhibitors with fewer side effects is crucial and urgent. In recent years, small molecule inhibitors have become a major research focus, including non-sulfonylurea compounds (e.g., representative compound B6) (Lv et al., 2025) and tetrahydroquinoline derivatives (Dai et al., 2021). These inhibitors interfere with NLRP3 assembly or activation by targeting NLRP3 directly or indirectly. Compared to MCC950, B6 exhibits higher potency and better tolerance (cell viability >95% at 500 μM concentration in human hepatocyte lines and mouse primary hepatocytes, whereas MCC950 showed <82% cell viability) (Lv et al., 2025). Additionally, peptide inhibitors exert therapeutic effects by modulating NLRP3 activation. Some inhibitors (e.g., SPA4 peptide) bind directly to TLR4 (a pathogen recognition receptor) during the priming phase, downregulating NF-κB activity and subsequently interfering with NLRP3 synthesis (Rakhesh et al., 2012). Innovative NLRP3 inhibition strategies extend beyond canonical targets through metabolic and epigenetic interventions. Metabolically, Vasoactive Intestinal Peptide (VIP) achieves redox homeostasis via NOX1/2 suppression (Sun et al., 2018) while concurrently activating cAMP-mediated negative feedback circuits (Zhou et al., 2020). Epigenetically, HDAC6 inhibition creates a multi-pronged effect: it transcriptionally represses NLRP3/IL-1β to attenuate inflammasome formation (Yan et al., 2020), while the inhibitor ACY1215 physically impedes activation through simultaneous regulation of F-actin dynamics and DDX3X function (Samir et al., 2019).

As a novel specific NLRP3 inflammasome inhibitor, OLT1177 offers several advantages over other inhibitors:

1. OLT1177 is an orally administered small molecule compound whose safety has been confirmed in human clinical trials (Chan et al., 2024). In Alzheimer’s disease (AD) models, oral administration of OLT1177 significantly improved cognitive dysfunction in recipients without observed serious side effects (Lonnemann et al., 2020). Furthermore, its safety profile has been validated in various disease models (e.g., Parkinson’s disease, ulcerative colitis) (Amo-Aparicio et al., 2023; Saber et al., 2021).

2. He antitumor efficacy of OLT1177 stems from its direct NLRP3 binding, which converges on STAT3 signaling suppression. In melanoma models, both monotherapy and dexamethasone combination therapy demonstrated significant tumor growth inhibition through coordinated blockade of STAT3 phosphorylation (Y705/S727) and consequent downregulation of nuclear and mitochondrial STAT3-dependent transcription (Dinarello et al., 2023). Notably, some other inhibitors (e.g., BBG) indirectly regulate NLRP3 by inhibiting P2X7R (Saber et al., 2021).

3. He ability of OLT1177 to cross the blood-brain barrier and reach effective concentrations translates into significant neuroprotection in PD models. Treatment prevented motor deficits through concerted actions centered on augmented microglial autophagy, which mediated α-synuclein clearance, attenuated neuroinflammation in nigrostriatal circuits, and conferred resilience to dopaminergic neurons facing MPTP toxicity (Amo-Aparicio et al., 2023).

4. OLT1177 has exhibited broad-spectrum therapeutic efficacy across diverse pathological contexts, demonstrating significant benefits in models of aging-related disorders (Muela-Zarzuela et al., 2024), metabolic disorders (Lonnemann et al., 2020), and oncological conditions (Dinarello et al., 2023). Compared to other NLRP3 inhibitors, OLT1177 has progressed to the clinical trial stage (Wohlford et al., 2020) and has shown good anti-inflammatory therapeutic effects in numerous disease models without significant observed side effects. However, despite its notable advantages, some studies suggest that monotherapy might have limited inhibitory effects on certain targets (e.g., NF-κB) (Saber et al., 2021), potentially necessitating combination with other drugs for optimal therapeutic outcomes. Furthermore, the long-term medication safety of OLT1177 requires further validation.

Although this review systematically elaborates on the mechanisms of action and therapeutic prospects of Dapansutrile, several limitations warrant attention in future research. First, the existing clinical evidence remains insufficient. Most of the efficacy data cited in this review are derived from preclinical animal models, while clinical studies of Dapansutrile in humans are predominantly in early stages (Marchetti et al., 2018b; Alenezi et al., 2025; Wohlford et al., 2020), characterized by limited sample sizes and short follow-up durations.Second, there is an inadequate understanding of the dose-response relationships across different diseases. This review indicates that Dapansutrile was used at significantly different dosage regimens in various disease models (e.g., gouty arthritis, Alzheimer’s disease (Marchetti et al., 2018a; Abbatecola et al., 2024)). A unified and optimized dosing strategy to achieve maximum efficacy and optimal safety across different pathological contexts has not yet been established, posing a challenge for its clinical translation.Third, investigation into the drug’s specificity requires further in-depth research. Although Dapansutrile is defined as a selective NLRP3 inhibitor, its potential cross-reactivity with other NLRP family members (such as NLRP1 or NLRP6) or the Pyrin inflammasome has not been fully elucidated.Fourth, long-term medication safety data are lacking. Reported adverse reactions are mild and transient in existing studies, but there is a lack of sufficient data to assess risks associated with long-term use over years or even decades, such as metabolic accumulation, organ toxicity, or immunosuppression. Finally, the mechanisms and optimization of combination therapy strategies need further exploration. Although this review mentions that Dapansutrile shows synergistic effects when combined with drugs like lonafarnib and dexamethasone (Muela-Zarzuela et al., 2024; Alenezi et al., 2025), the specific molecular networks and signaling pathway intersections underlying these synergistic actions remain unclear. How to select the optimal combination regimens and dosing sequences for different patient populations based on precision medicine principles is a key direction for future research.

In summary, although Dapansutrile, as a novel NLRP3 inhibitor, shows broad application prospects, the aforementioned limitations indicate a gap between mechanistic understanding and full clinical success, necessitating more in-depth and extensive research to bridge these cognitive gaps.

## 10 Conclusion

As a first-in-class specific inhibitor of the NLRP3 inflammasome, Dapansutrile (OLT1177) has shown considerable therapeutic promise across a diverse spectrum of disease models.In the field of acute kidney injury (AKI) (Elsayed et al., 2021), studies have confirmed its efficacy in suppressing NLRP3 inflammasome activation triggered by various etiologies including ischemia/reperfusion, folic acid induction, or hemolytic transfusion reactions. It alleviates renal tubular epithelial cell damage and renal dysfunction by reducing caspase-1 activation and GSDMD-NT generation12.For systemic inflammatory diseases, dapansutrile significantly improves sepsis-related organ damage and radiation-induced tissue injury by inhibiting mitochondrial damage-mediated NLRP3 activation3.Notably, the drug exhibits pleiotropic effects in progeria syndrome models: it not only reduces progerin accumulation and senescence-associated secretory phenotype (SASP), but also shows synergistic effects with FDA-approved lonafarnib, significantly extending model animal lifespan and improving kyphosis and other phenotypes (Muela-Zarzuela et al., 2024).

In tumor immunotherapy, dapansutrile enhances anti-PD-1 efficacy by modulating myeloid-derived suppressor cell (MDSC) expansion5 (Tengesdal et al., 2021). In Alzheimer’s disease models, it completely rescues memory deficits in APP/PS1 mice6 (Abbatecola et al., 2024; Lonnemann et al., 2020). With favorable oral bioavailability and an established safety profile in Phase II trials, Dapansutrile presents as a viable therapeutic candidate for clinical translation, particularly for NLRP3-driven conditions such as AKI and chronic inflammatory diseases where treatment options remain limited.
